# Frequency of Cry1F Non-Recessive Resistance Alleles in North Carolina Field Populations of *Spodoptera frugiperda* (Lepidoptera: Noctuidae)

**DOI:** 10.1371/journal.pone.0154492

**Published:** 2016-04-27

**Authors:** Guoping Li, Dominic Reisig, Jin Miao, Fred Gould, Fangneng Huang, Hongqiang Feng

**Affiliations:** 1 Department of Entomology, North Carolina State University, Vernon G. James Research and Extension Center, 207 Research Station Road, Plymouth, North Carolina, 27962, United States of America; 2 Henan Key Laboratory of Crop Pest Control, MOA’S Regional Key Lab of Crop IPM in Southern Part of Northern China, Institute of Plant Protection, Henan Academy of Agricultural Sciences, Zhengzhou 450002, the People’s Republic of China; 3 Department of Entomology, North Carolina State University, Raleigh, North Carolina, 27695, United States of America; 4 Department of Entomology, Louisiana State University Agricultural Center, Baton Rouge, Louisiana, 70803, United States of America; Pennsylvania State University, UNITED STATES

## Abstract

Fall armyworm, *Spodoptera frugiperda* (J.E. Smith) (Lepidoptera: Noctuidae), is a target species of transgenic corn (Zea mays L.) that expresses single and pyramided *Bacillus thuringiensis* (Bt) toxin. In 2014, *S*. *frugiperda* were collected from a light trap in North Carolina, and a total of 212 F_1_/F_2_ isofemale lines of *S*. *frugiperda* were screened for resistance to Bt and non-Bt corn. All of the 212 isolines were susceptible to corn tissue expressing Cry1A.105 + Cry2Ab, Cry1F + Cry1A.105 + Cry2Ab, and Cry1F + Cry1Ab + Vip3Aa20. Growth rate bioassays were performed to isolate non-recessive Bt resistance alleles. Seven individuals out of the 212 isofemale lines carried major non-recessive alleles conferring resistance to Cry1F. A pooled colony was created from the seven individuals. This colony was 151.21 times more resistant to Cry1F than a known-susceptible population and was also resistant to Cry1A.105, but was not resistant to Cry2Ab and Vip3Aa20. The results demonstrate that field populations of *S*. *frugiperda* collected from North Carolina are generally susceptible to Cry1F, but that some individuals carry resistant alleles. The data generated in this study can be used as baseline data for resistance monitoring.

## Introduction

Corn, *Zea mays* (L.), expressing the Cry1F protein (Event TC1507, Herculex^®^ I insect protection technology by Dow AgroSciences and DuPont Pioneer) was first registered in the United States during 2001. Many primary and secondary lepidopteran pest species, including fall armyworm, *Spodoptera frugiperda* (J.E. Smith) (Lepidoptera: Noctuidae) are targets of this event [1,2]. Field resistance of *S*. *frugiperda* to Cry1F corn was observed in Puerto Rico during 2006 and relatively high levels of Cry1F resistance were subsequently reported on this island following these initial observations [3,4]. Cry1F resistant *S*. *frugiperda* populations were documented on the U.S. mainland during 2011–2013 [5].

*S*. *frugiperda* is a widely distributed and well-known migrant pest of many crops throughout North and South America [6,7], and is a key pest of corn in the lower southeastern US [8]. It is also a sporadic pest of cotton, soybean and other crops, including many vegetables [9]. In North America, this insect has two distinct overwintering populations in Texas/Mexico and southern Florida that migrate north during spring [10,11]. These populations are separated by the Appalachian mountain range and overlap in Alabama/Georgia and in the mid-Atlantic [12,13]. Cry1F resistant *S*. *frugiperda* likely developed in 2006 in Puerto Rico [3]; although Cry1F resistant populations of *S*. *frugiperda* were not present on the U.S. mainland during 2012 [4], a 2013 study documented the first known Cry1F resistance in this geography. The highest Cry1F resistance ratios were located in Florida and North Carolina, but not in Georgia, Louisiana or Texas (excluding Louisiana and Florida lines isolated from populations using an F_2_ screen) [5]. In the U.S. mainland north of Florida, *S*. *frugiperda* has been effectively managed using corn hybrids expressing Cry1F [14,15]. The number of resistant and susceptible *S*. *frugiperda* individuals (frequency of resistant alleles) in the U.S. is unknown. With a migratory insect such as *S*. *frugiperda*, frequency of resistance in any location is likely influenced by fitness costs incurred by resistance [16], local selection, and influx of genes from immigrants.

To maintain the effectiveness of Bt corn, the frequency of resistance genes to Bt toxins should be monitored with a method that is appropriate for the pest and specific Bt crop [17,18]. Resistance evolution in field pest populations is complex and is influenced by a number of factors, including the extent of selection pressure exerted by Bt crops, the initial frequency of resistance alleles in the field population, and the migratory behavior of the adults [4,19]. Several methods have been developed to estimate recessive resistance frequencies to Bt corn in target insect pests [5,20–24]. Most methods focus on detecting the frequency of homozygous recessive Bt resistant individuals using specialized bioassays [25,26] and do not estimate the frequency of major non-recessive resistance alleles. Burd et al. [27] developed a bioassay to estimate the frequency of these major non-recessive resistance alleles using isofemale lines of F_1_/F_2_ generation. This method is appropriate when resistant alleles are not rare in the population.

One tactic that is important for maintaining insecticide resistance management is pyramiding Bt toxins, especially with pyramids that express proteins with dissimilar modes action, but that are effective against the same target pests [28]. Pyramided Bt corn hybrids (second generation) are being planted more frequently and are more effective for *S*. *frugiperda* management than single Bt protein hybrids (first generation) [14,15,28,29]. One reason pyramided Bt maize products are becoming more prevalent in North and South America is to manage Cry1F- resistant populations of *S*. *frugiperda*. Since resistance in North Carolina was detected during 2013 [5], we carried out experiments using the F_1_/F_2_ screening method that was proposed by Burd et al. [27] to monitor the frequency of non-recessive resistance alleles and larval susceptibility of *S*. *frugiperda* to Bt corn containing single or pyramided genes from eastern North Carolina, USA.

## Materials and Methods

### Bioassay of F_1_ generation on Bt and non-Bt corn tissue

During August through October 2014, a total of 400 *S*. *frugiperda* adult females were collected from a light trap at the Vernon G. James Research and Extension Center (N35.8750, W76.6606) in Plymouth, North Carolina, USA. Moths were individually placed into 350-ml clear plastic cups, covered with gauze, as oviposition substrate. Cups were kept at 27 ± 1°C, 70–80% RH, and L:D 14:10, and eggs were collected on a daily basis. If the moth produced more than 70 eggs daily, then the line was used in bioassays. We obtained sufficient eggs from 212 moths to use in bioassays.

The susceptibility of *S*. *frugiperda* was first evaluated on leaf tissue from two non-Bt and four Bt corn hybrids expressing single and pyramided traits (Table 1). Fully expanded leaf tissue of Bt and non-Bt corn hybrids was excised from greenhouse-grown V4–V10 stage plants. Pilot experiments indicated that larval response (growth and mortality) to Bt in leaf tissue of this age range was equivalent. Presence of Bt in the plants was confirmed using QuickStix kits (EnviroLogix, Portland, ME, data not presented), although each single transgenic leaf used in the study was not individually tested. In the bioassay, 2–3 pieces of leaf tissue from a single hybrid were placed in each well of a 32 well rearing tray (Frontier Agricultural Sciences, Newark, NJ). A single neonate (0–24 h old) was then placed into the well, with every *S*. *frugiperda* line tested on leaves from all corn hybrids. The total number of replicates for each *S*. *frugiperda* and hybrid combination was 32. Trays containing leaf tissues and neonates were placed in growth chambers maintained at 27°C, 70–80% RH, and L:D 14:10. After seven days, larval mortality was recorded, with larvae considered dead if they did not respond after being touched with a camel hair brush. The developmental stage of each surving larva was assessed using head capsule and body size as indicators; all growth stage values were converted to an ordinal ranking system where 0 = dead, 1 = first instar, 2 = early second, 3 = mid second, 4 = late second, 5 = early third, 6 = mid third, 7 = late third, 8 = early fourth, 9 = mid fourth. The weight of surviving larvae was also measured.

**Table 1:** Hybrids used to screen *S*. *frugiperda* in this experiment and the associated lepidopteran-specific Bt proteins.

Trade name	Hybrid	Lepidopteran-specific insertion event	Lepidopteran-specific Bt proteins
Non-Bt1	P1319R^a^	-	-
Herculex^®^I	P1319HR^a^	TC1507	Cry1F
Optimum^®^ Leptra^®^	P1319VYHR^a^	TC1507 + MON810 + MIR162	Cry1F + Cry1Ab + Vip3Aa20
Non-Bt2	DKC6482R^b^	-	-
Genuity^®^VT Double Pro^™^	DKC6489VT2P^b^	MON89034	Cry1A.105 + Cry2Ab
Genuity^®^ Smart-Stax^™^	DKC6487SS^b^	TC1507 + MON89304	Cry1F + Cry1A.105 + Cry2Ab2

^a^DuPont Pioneer, Johnston, IA

^b^Monsanto Company, St. Louis, MO

### Bioassay of F_2_ generation on Bt and non-Bt corn tissue

In order to determine the relationship between the F_1_ and F_2_ generation when feeding on Bt corn, F_1_ lines with a survivorship ≥50% were saved for testing during the F_2_ generation. F_1_ lines that developed ≥80% as well on Bt as they did on non-Bt corn were also saved for testing during the F_2_ generation. We hypothesized that F_1_ lines that developed relatively well on Bt corn carried at least one major non-recessive resistance allele in heterozygous form [30]. From each line, larvae that developed on non-Bt corn were reared to adult emergence and sib-mated. Resulting F_2_ neonates were subject to identical leaf tissue bioassays as described above. In this study, F_2_ larvae of 26 lines were tested using corn tissue expressing Cry1F, Cry1A.105 + Cry2Ab2 + Cry1F, and Cry1Ab + Cry1F + Vip3Aa20.

### Confirmation of resistance to Cry1F protein and other Bt protein resistance

The number of individuals with major resistance genes in a given population is expected to be low, and heterozygotes are the most probable carriers of resistance alleles in field populations [25]. Even if a female carries a major dominant resistance gene, the mean growth rate of her F_1_ offspring on corn expressing Bt should still be reduced compared to what it would be on non-Bt corn. Therefore, we assumed that if the relative average development ratings of any F_1_ line and the resulting F_2_ lines producted from those populations were ≥0.8 (rather than 1.0), and the corrected survival rate was ≥50% on Bt corn, then the line carried a major resistance gene [30].

Seven lines were sourced from the F_2_ screening that had a relative average developmental rating of ≥0.8 on leaves expressing Cry1F; these lines were then pooled in the F_3_ generation. The protein Cry1F was provided by Dow AgroSciences (Indianapolis, IN) as a gift and stored in a dessicator at -20°C. The proteins Cry2Ab2 and Cry1A.105 were provided by Monsanto Company as a gift and stored at -80°C. Finally, the protein Vip3Aa20 was provided by Syngenta Crop Protection (Greensboro, NC) and stored in a freezer at -20°C. Larval susceptiblitity of the pooled F_3_ line was assayed using a meridic diet overlay procedure in 128-cell plastic trays with a 2 cm^2^ surface area (Frontier Agricultural Sciences). The meridic diet (WARD’S Stonefly Heliothis diet, Rochester, NY) rearing procedure followed Niu et al. [29]. One ml of Heliothis diet was dispensed into each 2 cm^2^ well and allowed to solidify. In the bioassay, ten concentrations, plus a control, were serially diluted using CAPS buffer. Forty μl of the formulated solution or control was overlaid on the diet using a pipette; control treatments were composed of both distilled water and buffer. When all of the wells within a tray were treated, each tray was tilted from left to right and front to back to ensure that the liquid sample completely coated the surface of the diet. After no liquid was visible on the diet surface, one *S*. *frugiperda* neonate larvae (0–24 h old) was added to each well using a fine brush. The trays were sealed with self-adhesive plastic sheets (BIO-CV-16, CD International Inc.) and placed in a climatic chamber (27±1°C, 60±10% relative humidity, and L:D 14:10h). The bioassays were repeated three times for each population, with each concentration repeated three times per bioassay (total of three replications of 16 neonates/concentration). Mortality (with larvae that remained as first instars throughout the experiment also considered dead) and the weight of surviving larvae were measured at seven days after treatment as described before. A Bt-susceptible colony, of *S*. *frugiperda*, originally obtained from non-Bt maize in Hidalgo Co., TX (SS-TX) [5], was used as a control.

### Data analysis

To control for larval vigor effects, the growth rates of each line were compared on a Bt and a non-Bt hybrid from the same genetic background. Growth stage values were converted to an ordinal ranking system as described before. There were two hybrid groups: 1) P1319HR (Herculex^®^I), P1319VYHR (Optimum^®^ Leptra^®^), P1319R (related non-Bt, non-Bt1), and 2) DKC64-89 (Genuity^®^VT Double Pro^™^), DKC64-87 (Genuity^®^ Smart-Stax^™^), and DKC64-82 (related non-Bt, non-Bt2). The average developmental rating was defined as the the average ordinal ranking for a single female on each of the hybrids. The relative average developmental rating for a given line was calculated as the average developmental rating of a specific iso-female *S*. *frugiperda* line on given hybrid divided by the average developmental rating of the same iso-female *S*. *frugiperda* line on a given hybrid [31]. The corrected percentage survival was obtained using the Abbott’s formula [32]. Pearson’s correlation analysis was used to compare the average developmental rating, the mass weight and the survival rate of a given iso-female line to Bt and non-Bt corn hybrids, or to compare the relative average developmental rating and corrected survival rate of larvae of a given F_1_ generation with the F_2_ generation [33]. LC_50_ (GIC_50_) or LC_90_ values were obtained and compared using POLO-Plus [34], with significance defined as non-overlapping confidence limits.

## Results

### Bioassays on F_1_ generation test

**Fig 1:**
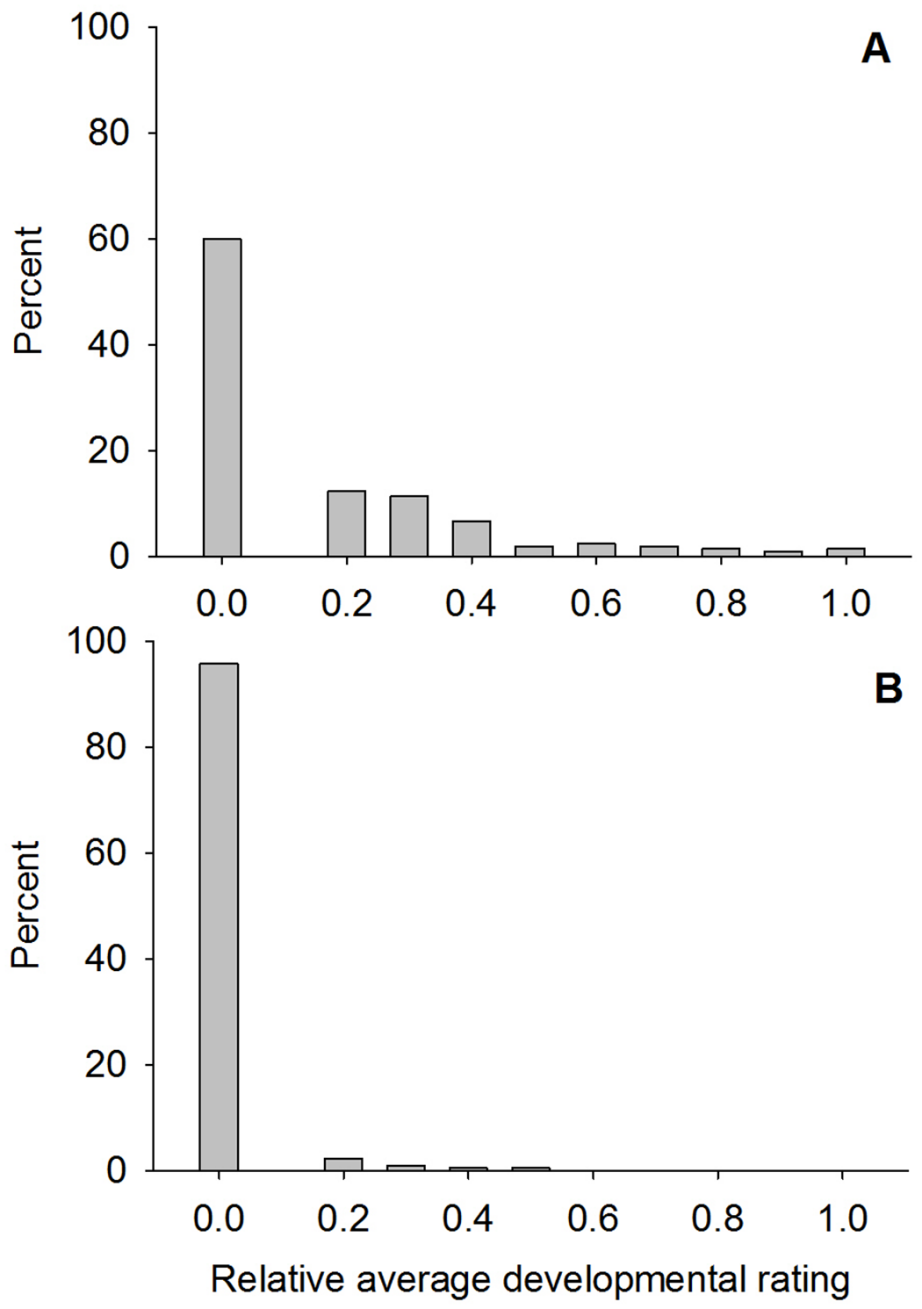
Distribution of the relative average development rating for seven-day old F_1_ larvae of S. *frugiperda* female lines on corn leaf tissue expressing Cry1F (A) or Cry1A.105 + Cry2Ab (B).

A total of 212 female lines (isofemales) were screened on leaves expressing Cry1F. The relative average developmental ratings for most lines on Cry1F ranged from 0.00 (dead) to 1.00 with a mean relative average developmental rating of 0.15 (Fig 1a, Table 2). Twenty-six out of 212 lines had a corrected survival rate ≥50% and were saved for testing the F_2_ generation (Table 2). Among the 26 lines, there were eight lines with a relative average developmental rating ≥0.80 (Table 2).

**Table 2:** Estimated resistance allele frequency for major non-recessive resistance alleles with Bayesian 95% credibility intervals for field-collected *S*. *frugiperda* individual female lines (*n* = 212) to Bt corn hybrids.

Protein expressed in leaf tissue	F_1_ generation bioassay				F_2_ generation bioassay			Estimated r allele frequency (95% CI)^b^
	Number tested	Relative average development rating per line Mean (±SE)	% Corrected survival ≥50%	Number with relative average developmental rating ≥0.8	Number tested	Relative average development rating per line Mean (±SE)	Number with relative average developmental rating ≥0.8^a^	
Cry1F	212	0.15 ± 0.02	26	8	26	0.67 ± 0.03	7	0.009346 (0.004048–0.016795)
Cry1A.105 + Cry2Ab	212	0.01 ± 0.00	0	0	-	-	-	<0.001168 (0.00003–0.004305)
Cry1F + Cry1A.105 + Cry2Ab2	212	0.00 ± 0.00	0	0	26	0.00 ± 0.00	0	<0.001168 (0.00003–0.004305)
Cry1F + Cry1Ab + Vip3Aa20	212	0.00 ± 0.00	0	0	26	0.00 ± 0.00	0	<0.001168 (0.00003–0.004305)

^a^Moth was regarded as an individual with a resistance allele if, during the F_1_ and F_2_ generation, the relative average development ratings were ≥0.8.

^b^Each mated female carries two of her own alleles and two from her male counterpart (if she mated only once); by screening females on Bt corn to characterize (4 × total number) the genome, the estimated frequency for the resistance allele to each Bt hybrid would be: (the number of individual with resistance allele + 1) / 4 × (total number + 2)[26].

A total of 212 female lines (isofemales) were screened on leaves expressing Cry1A.105 + Cry2Ab2. The relative average developmental ratings for most lines on Cry1A.105 + Cry2Ab2 ranged from 0.00 (dead) to 0.50 with a mean relative average developmental rating of 0.01 (Fig 1b, Table 2). None of the 212 female lines had a relative average developmental rating ≥0.80 or a corrected surviorship ≥50% (Table 2). No larvae survived on leaves expresing Cry1F + Cry1A.105 + Cry2Ab2 or Cry1F + Cry1Ab + Vip3A; hence, relative average development ratings were 0.00 for these Bt pyramids.

Average development ratings were not correlated between leaves expressing Cry1F and leaves from a related non-Bt hybrid, or between leaves expressing Cry1A.105 + Cry2Ab2 and leaves from a related non-Bt hybrid (Table 3). Similarly, relative average development ratings were not correlated between leaves expressing Cry1F and leaves expressing Cry1A.105 + Cry2Ab2. Furthermore, there was no correlation for survival between leaves expressing Cry1F, or Cry1A.105 + Cry2Ab2, and leaves from their respective related non-Bt hybrids; finally, there was no correlation between corrected survival between leaves expressing Cry1F and leaves expressing Cry1A.105 + Cry2Ab2 (Table 3).

**Table 3:** Pearson correlation values of average developmental ratings, relative average developmental ratings, percent survival (corrected using Abbot’s formula [32]), and larval weight (after seven days) of F_1_ *S*. *frugiperda* lines selected on leaves expressing Bt and non-Bt leaves of closely related hybrids. * = *P* <0.05, ** = *P* <0.001.

Correlation	*r*	*P*
Average developmental rating on non-Bt1 and Cry1F	0.0142	0.8374
Average developmental rating on non-Bt2 and Cry1A.105 + Cry2Ab2	-0.0526	0.4465
Relative average developmental rating on Cry1F and Cry1A.105 + Cry2Ab2	0.0962	0.1629
Percent survival on non-Bt1 and Cry1F	0.0614	0.3735
Percent survival on non-Bt2 and Cry1A.105 + Cry2Ab2	0.0641	0.3529
Percent corrected survival on Cry1F and Cry1A.105 + Cry2Ab2	-0.0201	0.7715
Percent survival and average developmental rating on non-Bt1	0.0781	0.2577
Percent survival and average developmental rating on Cry1F	0.7522**	<0.0001
Percent survival and average developmental rating on non-Bt2	0.1533*	0.0256
Percent survival and average developmental rating on Cry1A.105 + Cry2Ab2	0.7518**	<0.0001
Larval weight on Cry1F and Cry1A.105 + Cry2Ab2	-0.0379	0.5828
Percent survival and larval weight on non-Bt1	0.2516**	0.0002
Percent survival and larval weight on Cry1F	0.5402**	<0.0001
Percent survival and larval weight on non-Bt2	0.5310**	<0.0001
Percent survival and larval weight on Cry1A.105 + Cry2Ab2	0.8047**	<0.0001
Average developmental rating and larval weight on non-Bt1	0.4552**	<0.0001
Average developmental rating and larval weight on Cry1F	0.4986**	<0.0001
Average developmental rating and larval weight on non-Bt2	0.3224**	<0.0001
Average developmental rating and larval weight on Cry1A.105+ Cry2Ab2	0.8808**	<0.0001

There was a correlation between average developmental rating and survival on leaves expressing Cry1F, larval weight and survival on leaves expressing Cry1F, and average development rating and larval weight on leaves expressing Cry1F (Table 3). In contrast, there was no correlation between average development rating and survival on non-Bt leaves from the same hybrid family as the Cry 1F leaves (non-Bt1). Larval weight was correlated with survival, as was larval weight and average developmental rating, on non-Bt leaves from the same hybrid family as the Cry1F leaves (non-Bt1).

Average developmental rating was correlated with survival, as well as larval weight on leaves expressing Cry1A.105 + Cry2Ab2 (Table 3). Similarly, these factors were correlated for larvae that developed on non-Bt corn leaves from the same hybrid family as the Cry1A.105 + Cry2Ab2 leaves (non-Bt2).

### Bioassay on F_2_ generation test

**Fig 2:**
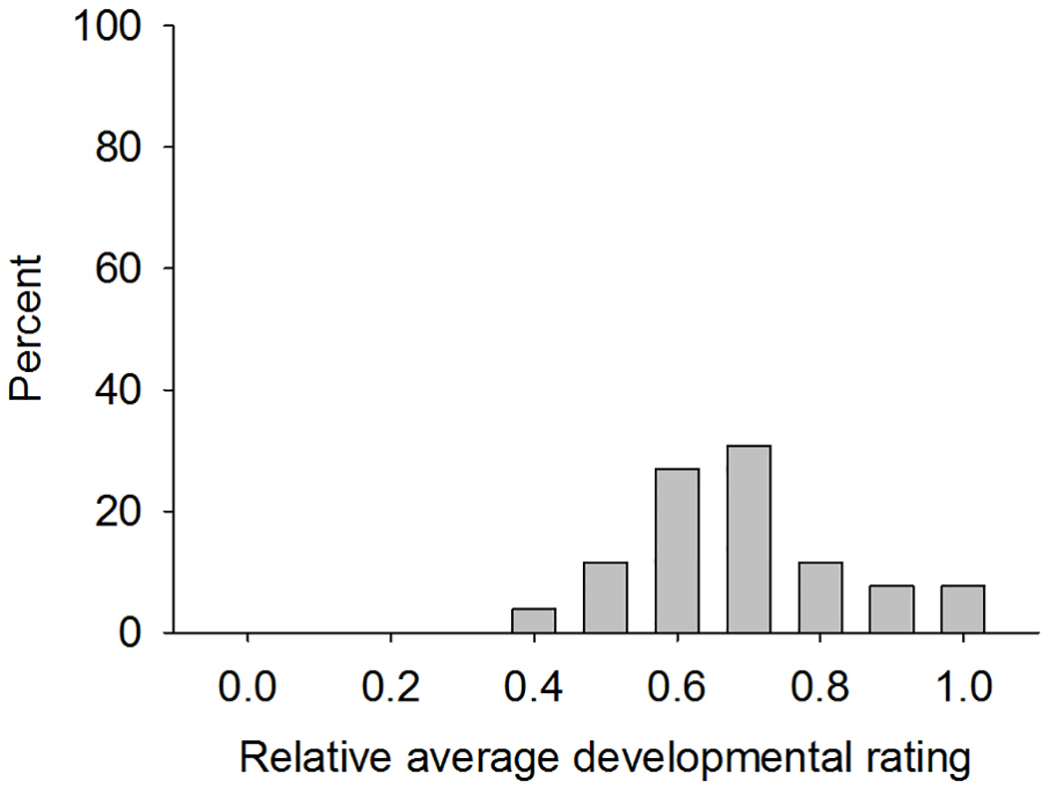
Distribution of the relative average development rating for seven-day old F_2_ larvae of *S*. *frugiperda* female lines on corn leaf tissue expressing Cry1F.

A total of 26 isofemale lines that survived to the F_2_ generation were screened on corn leaves expressing Cry1F, Cry1F + Cry1A.105 + Cry2Ab2, and Cry1F + Cry1Ab + Vip3A20. The distribution of the relative average developmental ratings on Cry1F leaves of seven-day old larvae from all these lines on Cry1F is presented in Fig 2. These relative average development ratings for most lines on Cry1F ranged from 0.40 to 1.0 with a mean relative average development rating of 0.67 (Table 2). No larvae survived on Cry1F + Cry1A.105 + Cry2Ab2 and Cry1F + Cry1Ab + Vip3A; consequently relative average development ratings were 0.00.

**Fig 3:**
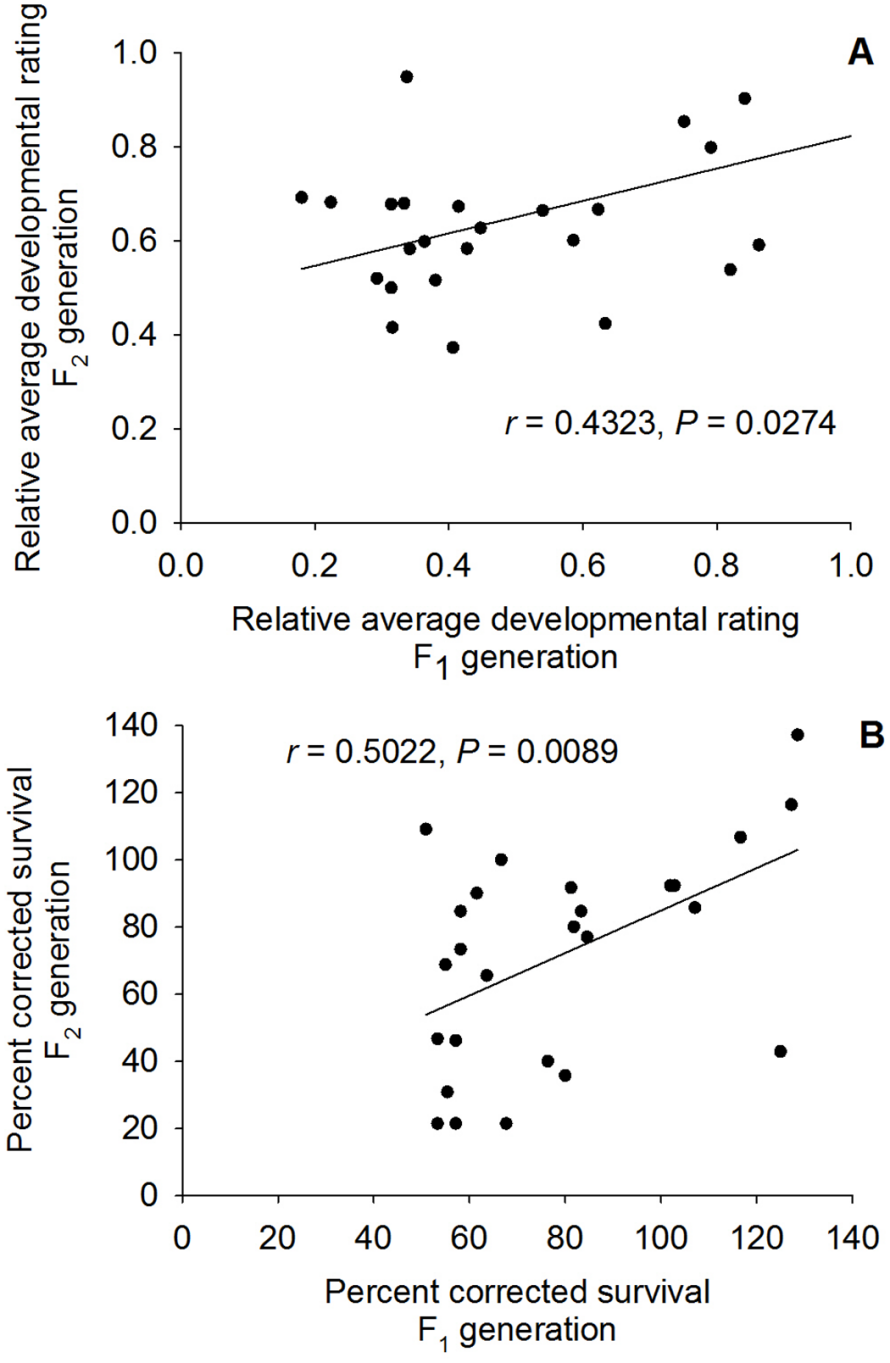
Correlations between the relative average developmental ratings (A) and corrected percent survival (B) for *S*. *frugiperda* lines during the F_1_ generation and their offspring during the F_2_ generation. Larvae were reared on corn tissue expressing Cry1F. Pearson correlation results reported as *r* and *P* values.

There was a positive correlation between relative average development ratings of these 26 lines between the F_1_ and F_2_ generation on Cry1F leaves (*r* = 0.4323, *P* = 0.0274, Fig 3a). There was also a positive correlation between corrected seventh day survival of these 26 lines between the F_1_ and F_2_ generation on Cry1F leaves (*r* = 0.5022, *P* = 0.0089, Fig 3b). Since these results indicate a genetically-based variation in response to Cry1F, the frequency of major resistance alleles to this protein was calculated as 0.009346 (Table 2).

### Confirmation of resistance to Cry1F protein and other Bt protein resistance

**Fig 4:**
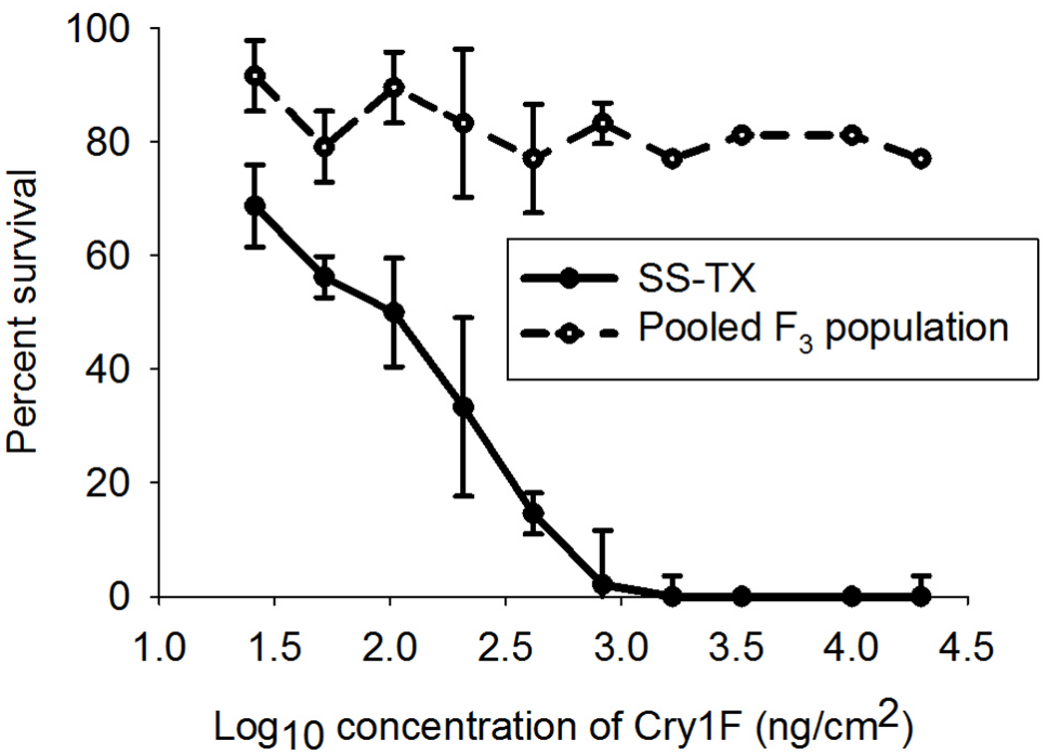
Concentration-mortality response (ng/cm^2^) of *S*. *frugiperda* neonates of a known susceptible population (SS-TX) and a population selected on Cry1F corn leaf tissue (pooled F_3_) to a diet overlay assay using Cry1F protein.

There were seven F_2_ generation lines with relative average developmental ratings ≥0.8 on Cry1F leaf tissue; this relative average developmental rating was used as a threshold, above which the development of these larvae on Cry1F leaf tissue was considered to be substantially higher compared to other lines in the F_2_ generation (Table 2). The seven lines with a relative average developmental rating ≥0.8 on Cry1F leaves in the F_2_ generation were pooled, mated, and tested for their resistance level to Cry1F, Cry2Ab2, Cry1A.105, and Vip3Aa20 protein during the F_3_ generation (pooled population). The corrected percent surviorship after seven days of a Cry1F-susceptible control line (SS-TX) was zero at 1667 ng/cm^2^, while the corrected surviorship after seven days of the pooled-population was 78.72% at the same concentration of protein (Fig 4). The LC_50_ of the SS-TX population to Cry1F was 131.601 ng/cm^2^. No significant larval mortality (≤21.28%, corrected mortality) of the pooled population was observed across any of the Cry1F concentrations assayed; thus, the LC_50_ value of this population was estimated to be greater than the highest Cry1F concentrations tested (>19900 ng/cm^2^), which corresponded to a resistance ratio of >151.21.

The LC_50_ of the SS-TX population to Cry1A.105 was 0.241 ng/cm^2^, 4.4 times lower than that of the pooled population (1.070 ng/cm^2^). Since the 95% confidence limits overlapped, this difference was not significant. In contrast, the confidence limits of the LC_90_ values did not overlap. The LC_90_ of the SS-TX population was 30.5 times lower (1.292 ng/cm^2^) than that of the pooled population (39.382 ng/cm^2^, Table 4). The growth inhibition curve after seven days (GIC_50_), was 0.027 ng/cm^2^ with 95% CL of 0.004–0.072 for the SS-TX population and 0.880 ng/cm^2^ with 95% CL of 0.306–3.426 for the pooled population. This indicates that the pooled population was 32.59 times more resistant to Cry1A.105 compared to the known Cry1F susceptible control population.

**Table 4:** Concentration-mortality response (ng/cm^2^) of *S*. *frugiperda* neonates of a known susceptible population (SS-TX) and a population selected on Cry1F corn leaf tissue (pooled F_3_) to diet overlay assays using Cry1F, Cry2Ab2, Cry1A.105 and Vip3Aa20 proteins. SE = standard error; CL = confidence limits; LC_50_ = concentration of protein (ng/cm^2^) required to kill 50% of larvae in the observation period of seven days; LC_90_ = concentration of protein (ng/cm^2^) required to kill 90% of larvae in the observation period of seven days.

Protein	Population	*n*	Slope ± SE	LC_50_ (95% CL)^a^	LC_90_ (95% CL)^a^
Cry1F	SS-TX	336	1.986 ± 0.370	131.601 (66.591–193.520)	581.459 (417.808–950.041)
	Pooled F_3_	512	0.146 ± 0.088	>19900	-
Cry2Ab2	SS-TX	336	1.851 ± 0.441	160.351 (35.346–263.473)a	790.135 (491.267–3178.453) a
	Pooled F_3_	336	1.135 ± 0.401	408.462 (93.176–836.623)a	5487.257 (1924.345–906567.387)a
Cry1A.105	SS-TX	336	1.759 ± 0.410	0.241 (0.079–0.420)a	1.292 (0.781–2.951)a
	Pooled F_3_	336	0.818 ± 0.181	1.070 (0.205–3.144)a	39.382 (9.896–2456.091)b
Vip3Aa20	SS-TX	336	2.328 ± 0.469	156.496 (87.152–217.960)a	555.817 (404.762–945.182)a
	Pooled F_3_	336	1.376 ± 0.235	33.913 (6.172–71.768)b	289.402 (145.516–1015.551)b

^***a***^Values designated by different letters within a column are significantly different from each other. Values were significant when 95% fiducial limits did not overlap.

The LC_50_ and LC_90_ response to Cry2Ab was not different between the SS-TX and pooled populations. A significant difference in mortality response was observed to Vip3Aa20 between the two populations in both the LC_50_ and the LC_90_ (Table 4); the pooled population was more sensitive to Vip3A20 than SS-TX population (sensitivity ratio = 4.61).

## Discussion

In this study, we used the F_1_/F_2_ larvae of isofemale lines to test the frequency of non-recessive resistance alleles of *S*. *frugiperda* collected in North Carolina. The bioassays of F_1_/F_2_-generation individuals used in this study were specifically designed to test the fitness of individuals with resistance genes, to detect the presence of dominant resistance genes in the heterozygous form, or to detect the presence of recessive resistance genes in the homozyous form. A total of 212 isofemale lines were successfully screened on leaves expressing Cry1F; of these, seven individuals were identified as carrying major alleles conferring resistance to Cry1F. The major resistance allele frequency to Cry1F was 0.009346. According to the categories and patterns of field-evolved resistance to Bt crops described by Tabashink et al. [35], the resistance level of *S*. *frugiperda* to Cry1F in this location could be characterized as an early warning of resistance, as 3% of the individuals were resistant, with good development on Cry1F corn leaves compared to non-Bt. The relatively low frequency of resistance alleles in this single location also confirmed the observations of previous investigators, who characterized corn hybrids expressing Cry1F as effective to manage *S*. *frugiperda* in the mainland US north of Florida [14, 15]. It should be noted that this estimation is conservative because it did not estimate the frequency of recessive alleles in the heterozygous form.

In addition, we did not detect resistance to Bt corn pyramids expressing Cry1A.105 + Cry2Ab, Cry1F + Cry1A.105 + Cry2Ab, and Cry1F + Cry1Ab + Vip3Aa20. Pyramided Bt crops can delay the evolution of resistance by producing two or more distinct toxins that kill the same pest, although the effect of the pyramid is reduced when there is cross resistance [36] and there is resistance to one or more of the toxins in the pyramid [37]. Our findings of increased lethal concentration values to Cry1A.105 protein in the population selected on Cry1F leaves (F_3_ pooled population) compared to a known Cry1F susceptible population (SS-TX) indicate that, based on the current available pyramided Bt corn hybrids, pyramiding without consideration for cross-resistance may not be the best tactic to delay the development of resistance. However, we did not clearly show cross resistance, as there were no correlations between Cry1F and Cry1A.105 + Cry2Ab2 for relative average developmental ratings, percent survival, and larval weight (after seven days) of F_1_ *S*. *frugiperda* lines. Furthermore, diverse mechanisms of resistance to Bt toxins and diverse mutations of resistance alleles associated with Bt toxins have been reported for many insect species [38–43]. Hence, the genetic basis for resistance to pyramided Bt crops may involve multiple loci. As a result, the method proposed by Andow and Alstad [26] to estimate the frequency of resistance may overestimate this frequency, since it is based on the assumption of a single resistant allele. A appropriate method should be developed to estimate the frequency of resistant alleles to Bt crop pyramids in the future.

The most probable carrier for a resistance gene in field populations is a heterozygote, since homozygous resistant individuals are rarer. Also the most probable mating is between heterozygote and homozygote susceptible individuals [44]. Thus, the most likely offspring from this cross would be one-half heterozygote and one-half homozygote susceptible. Hence, the Cry1F resistance gene we observed is dominant or incompletely dominant, since 26 out of the 212 isofemale lines had a corrected survival ≥ 50% and relative average developmental rating ≥ 0.8 on Cry1F leaves in the F_1_ generation. Results in this paper are not consistent with those reported previously [3,18,23], which indicated that the Cry1F resistance gene of *S*. *frugiperda* in Puerto Rico was recessive or incompletely recessive. Likely the inheritance of resistance to Cry1F in populations of *S*. *frugiperda* is complex.

In our study, resistance was characterized by F_1_/F_2_ screening using corn leaves expressing Cry1F. After the F_2_ generation, seven resistant individuals were pooled and their offpsring were assayed in the F_3_ generation. These displayed a >151.21-fold resistance to Cry1F compared to a known susceptible population. Based on the growth inhibition bioassay, the selected pooled population also displayed a 32.59-fold resistance to Cry1A.105. These results are consistent with a previous study which demonstrated resistance to both Cry1F and Cry1A.105 in a Florida *S*. *friguperda* population [5]. Since both Cry1F and Cry1A.105 have a high affinity and compete for the same binding sites, cross-resistance is likely between these two proteins [45]. Furthermore, Cry1F-resistant *S*. *frugiperda* was susceptible to Cry2Ab and Vip3Aa20, consistent with populations tested from Florida [5] and Puerto Rico [23]. Since these proteins do not share midgut binding sites, cross-resistance among these toxins would be unexpected [23,45,46]. However, the population selected on Cry1F leaves (pooled population in this study) was more susceptible to Vip3Aa20 than a known Cry1F susceptible population (SS-TX). The explanation for this finding is unclear. It is possible that there is antagonism or negative cross-resistance between Cry1F and Vip3Aa20. Another possibility is that there is inherent variation in susceptibility to Vip3Aa20 between these two strains that are from two geographically distinct and genetically distinct populations [13].

Nonetheless, our results also suggest that deploying Cry2Ab and Vip3Aa20 alone or in a pyramid is an effective tactic to manage *S*. *frugiperda* in the southern US. Moreover, the high frequency of Cry1F resistant *S*. *frugiperda* populations in Puerto Rico, the tropical climate, the year-round cultivation of maize, extensive prior use of Bt as an insecticidal foliar spray, abundant pest populations, drought conditions, and minimal use of non-Bt refuge are likely the main factors that led to resistance evolution in *S*. *frugiperda* [3,4, 24]. Most of these conditions are not present in the North Carolina environment, with the exception of short-term drought and minimal use of non-Bt refuge. Huang et al. [5] speculated that because *S*. *frugiperda* is a polyphagous insect with a wide host range, selection pressure in North Carolina does not appear to be a major factor driving the development of field resistance. In our North Carolina study, we documented resistance allele frequency to Cry1F as 0.009346. It is unclear whether this resistance is a result of immigrants from other areas or from local selection in North Carolina. More work should be done to document the host range of this insect and the interplay of local movement and long-range dispersal to improve resistance management in Bt crops.
